# The importance of sampling standardization for comparisons of insect herbivory in deep time: a case study from the late Palaeozoic

**DOI:** 10.1098/rsos.171991

**Published:** 2018-03-28

**Authors:** Sandra R. Schachat, Conrad C. Labandeira, S. Augusta Maccracken

**Affiliations:** 1 Department of Paleobiology, Smithsonian Institution, Washington, DC 20013, USA; 2 Department of Geological Sciences, Stanford University, Stanford, CA 94305, USA; 3 Department of Entomology, University of Maryland, College Park, MD 20742, USA; 4 College of Life Sciences, Capital Normal University, Beijing 100048, People's Republic of China

**Keywords:** ecology, folivory, gymnosperm, Permian, plant–insect interactions, rarefaction

## Abstract

Sampling standardization has not been fully addressed for the study of insect herbivory in the fossil record. The effects of sampling within a single locality were explored almost a decade ago, but the importance of sampling standardization for comparisons of herbivory across space and time has not yet been evaluated. Here, we present a case study from the Permian in which we evaluate the impact of sampling standardization on comparisons of insect herbivory from two localities that are similar in age and floral composition. Comparisons of insect damage type (DT) diversity change dramatically when the number of leaves examined is standardized by surface area. This finding suggests that surface area should always be taken into account for comparisons of DT diversity. In addition, the three most common metrics of herbivory—DT diversity, proportion of leaves herbivorized and proportion of leaf surface area herbivorized—are inherently decoupled from each other. The decoupling of the diversity and intensity of insect herbivory necessitates a reinterpretation of published data because they had been conflated in previous studies. Future studies should examine the divergent ecological factors that underlie these metrics. We conclude with suggestions to guide the sampling and analysis of herbivorized leaves in the fossil record.

## 1. Introduction

Plant–insect interactions are essential to the foundation of terrestrial ecosystems and have a documented evolutionary history that extends at least to the Middle Devonian, approximately 385 million years before the present [1]. Two principal types of evidence yield insights into the coevolution of plants and insects. First is the insect body fossil record, which includes specialized feeding and oviposition structures involved in interactions with plants [2]. The second line of evidence is the plant fossil record, which includes damage caused by herbivorous insects, such as hole feeding, leaf mining or skeletonization [3]. With complete datasets collected from all plant specimens at an entire locality, this fossil record can also be used to track changes in insect herbivory over time, by comparing either the amount of leaf area herbivorized or the diversity of modes of herbivory across various floras. This comparative approach has been used for series of floras within specific regions and time intervals [4,5] and, in a recent contribution, across the fossil record [6].

The study of insect herbivory was only recently formalized [7], and a main issue in palaeobiology, sampling standardization, has only recently been addressed in this field [8]. When attempting to reconstruct the taxonomic diversity of animal life—particularly for marine invertebrates—palaeobiologists have rigorously tested an array of subsampling approaches with the aim of correcting for biases in the fossil record such as collecting effort, depositional environment and anatomy [9]. Different intervals and localities have been sampled with varying degrees of intensity, and the dearth of data for certain intervals must be taken into account in order for comparisons of diversity across space and time to reflect true biological patterns. However, when insect herbivory has been compared across fossil localities, the issue of incomplete sampling has not been fully addressed. In previous contributions, sampling standardization was conducted for individual metrics of herbivory: damage type (DT) diversity [10,11], functional feeding group (FFG) diversity [12] and the proportion of leaf area damaged by insects [13], also known as the herbivory index. However, confidence intervals have not been presented using leaf-area measurements. In many other comparisons, raw data were used [5,6]. Some of these analyses were conducted on a restricted dataset of broadleaf taxa only—in order to control for differences in the proportion of seeds, axes and other plant organs—but the variation in the number of broadleaf specimens examined was not accounted for in any way.

The present contribution aims to: (i) assess the impact of sampling standardization on comparisons of insect herbivory across multiple localities; (ii) explore correlations between different measures of insect herbivory; and (iii) establish guidelines for sampling standardization in future studies.

## 2. Background

### 2.1. Sampling of fossil leaves

A key concern in deep-time plant–insect associational studies involves comparisons of publications with disparate collection and sampling methods [14,15]. Such collections may contain sampling biases that could skew herbivore diversity and abundance inferences due to a disproportionate representation of rare, tough and pristine leaves [16]. ‘Cherry picking’ is the practice of preferentially collecting leaves that are rarely encountered and leaves with well-preserved features undamaged by insect herbivores. These collecting biases create palaeobotanical collections skewed towards rare and robust plant taxa [8,17,18]. Collecting and counting only leaves that are at least 50% complete ensures that each leaf is counted only once [18]. Plant identification is also substantially more reliable when over half of the leaf is intact [19]. This sampling method may result in bias against heavily damaged leaves or rare taxa for which specimen completeness cannot be assessed. At the other extreme is the collection of all complete plant specimens and fragments of plant macrofossil that measure at least 0.5 cm^2^ in area [14,17,20,21]. This approach precludes any bias against damaged leaves and permits the identification of all herbivore interactions. However, this procedure makes leaf identification difficult and allows multiple fragments of the same leaf to be counted. Notably, identification is relatively straightforward for gymnosperm-dominated floras [22–25] but far more difficult for angiosperm-dominated floras, which are typically more diverse. Unlike a field census, a complete sampling routine—in which all leaf fossils are collected—will permit replicable, unbiased studies.

Sampling practices among deep-time plant–insect associational studies also vary. In most Palaeozoic, Triassic and Jurassic research, all plant fragments greater than 0.5 cm^2^ in surface area and all complete plant organs were included [5,26–29]. The same practice has also been used with a minimum size requirement of 1 cm^2^ in at least one Cenozoic study [30]. This method may overestimate the abundance of specimens and/or insect damage occurrences because it is possible to count a single, fragmented leaf more than once. Surface area measurements overcome the obstacle of multiple counts, but are labour-intensive. Cretaceous and Cenozoic studies have used a wider variety of sampling methods on existing palaeobotanical collections. These methods range from collecting data only on leaves that are at least 50% complete [4,31,32], to identifiable and non-fragmentary leaves [33], to any leaf with identifiable insect-mediated damage [34]. Most Mesozoic and Cenozoic studies do not include surface area data. Recent studies have compared plant–insect interactions at disparately collected and sampled assemblages [6,13], underscoring the need for uniform sampling standardization procedures.

### 2.2. Subsampling methodology

Rarefaction methods can be used to compute either richness or density and can be implemented as either individual-based or sample-based rarefaction [35]. Richness is typically of interest in addressing theoretical issues and in model testing [35], whereas density measures DT diversity for a given amount of foliage. Furthermore, sample-based measures of density are not biased by DT evenness. For these reasons, DT density as measured by sample-based rarefaction is the more appropriate metric to be used in comparisons of insect herbivory across different localities and has been used in previous studies either exclusively [19,31,33] or alongside individual-based rarefaction, in which each occurrence of damage is treated as an ‘individual' [13]. Here, ‘diversity’ is used as a synonym for ‘density' as measured by sample-based rarefaction. We focus on density because this measure accounts for sampling effort, and here we examine the importance of standardizing for sampling effort when comparing DT diversity.

DT density can be determined through either individual-based rarefaction, in which DTs are sampled individually and the total number of DTs seen is tallied after each individual DT is sampled, or by sample-based rarefaction, in which leaf specimens are sampled individually and the total number of DTs seen is tallied after each specimen is sampled. Sample-based rarefaction allows standardization of the number of leaves sampled per site, so this method is most commonly used [19,31,33]. Furthermore, sample-based rarefaction is usually the only method that can be used in studies of fossil herbivory, because individual-based rarefaction requires abundance data [35] but DT occurrences have historically been tallied simply as presence/absence data for each leaf specimen.

**Figure 1.**
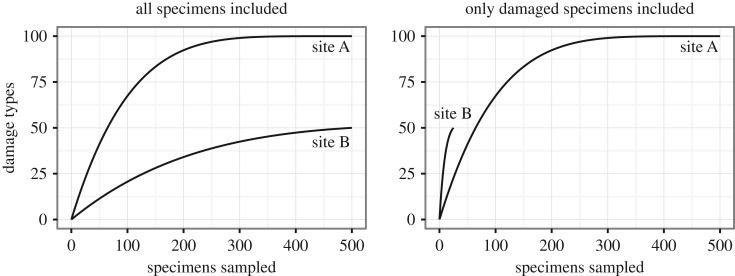
Rarefied DT diversity from two hypothetical datasets, with and without undamaged leaves included as ‘samples'. See text for details about the composition of each dataset.

For these reasons, sample-based rarefaction is used to measure DT density. Previous studies have typically treated all leaf specimens, with and without insect damage, as ‘samples’ [19,31,33,36]. The exclusion of undamaged leaves would bias the dataset, artificially inflating the prevalence of herbivory and possibly exerting varying amounts of bias at different sites. This issue can be illustrated by a hypothetical example in which two sites both contain 500 leaves each, both with the same amount of foliar surface area. The leaves from Site A have a total of 100 DTs, with each DT occurring on five leaves and with no single leaf containing more than one DT. The leaves from Site B have a total of 50 DTs: 25 leaves have a random assortment of five DTs each, and the remaining 475 leaves do not have any insect damage. If all damaged and undamaged leaves are included as ‘samples’ in the rarefaction analysis, the resulting rarefaction curves correctly show that Site A has a greater density of DTs than Site B (figure 1). However, if the only leaves included as ‘samples' in the rarefaction analysis are those with insect damage, the resulting rarefaction curves are problematic for two reasons. Firstly, only 50 samples are included from Site B, so sampling effort incorrectly appears to have been unequal for the two sites. And secondly, the rarefaction curves cannot be compared beyond the level of 25 specimens, and at this level Site B incorrectly appears to have a greater DT density than Site A (figure 1).

Wilf *et al*. [36] computed DT diversity rarefaction curves with and without undamaged specimens. They found that exclusion of the undamaged specimens led to a reduction in noise. However, the inclusion of undamaged specimens may lead to a reduction in precision (i.e. confidence intervals will be wider) but will provide greater accuracy (i.e. the resulting mean and confidence interval will be more likely to reflect the true value).

Traditionally, rarefaction has been used for neontological studies in which living species are tallied across different sites [35,37]. In such studies, there may be no *a priori* criterion for the inclusion of samples, or sites, and if a certain site does not yield any of the species of interest, then the site will be excluded from further analyses. Studies of DT diversity use the same rarefaction methods that are typically used by neontologists, with the most obvious differences being that leaf specimens are treated as analogues of sites and DTs are treated as analogues of species. However, another difference between neontological ecology and the study of DTs in the fossil record is that studies of DT diversity do have an *a priori* criterion for the inclusion of samples: all plant specimens from a given fossil locality are relevant samples. While the exclusion of certain samples may be a necessity for rarefaction curves based on neontological data, this necessity does not exist for studies of DT diversity, and the exclusion of undamaged leaf samples can bias the resulting rarefaction curves.

### 2.3. Diversity versus intensity of herbivory

The intensity of herbivory and the diversity of herbivore-induced DTs are frequently used to gauge the extent of plant–insect associations at a given site [4,38,39]. In previous studies, these two measures have been conflated and used interchangeably [4,19,30,38], despite the fact that they measure different ecological phenomena. The intensity of herbivory is often measured with one of two metrics: the proportion of leaves exhibiting insect damage or the proportion of total surface area removed by insect herbivores. In both measurements, the level of intensity may vary depending on various factors: the frequency with which phytophagous insects target a particular plant [40]; the defences (constituent or induced) that the plant employs [41]; the potency of those defences [42]; plant tolerance [43,44]; the location of a particular leaf on the plant [45]; the age of the leaf [46,47]; the location of the plant in relation to other plant species [48]; the abundance of insect herbivores [49]; and the effect of climate and atmospheric composition, such as temperature and carbon dioxide levels, respectively, on plant metabolism [50,51]. The diversity of DTs reflects the diversity of feeding modes (feeding guilds coupled with feeding pattern behaviours) at a locality. DT diversities may be influenced by the variation in feeding modes between insect species [52], the variety of DTs that a single individual may produce [52], floral diversity and the abundance of each plant species [53], and whether the plant attracts more generalist or specialist insects. A plant that only attracts specialist insects is less likely to exhibit evidence of a wide range of feeding modes [54].

The intensity at which a community of insects feeds upon a particular flora may have a positive relationship, a negative relationship or no relationship to the diversity of DTs found on the flora. There may be an intrinsic relationship between intensity of herbivory and diversity of DTs, in that higher DT diversity will lead to higher intensity of herbivory if the frequency of each DT remains constant as the total number of DTs changes [55]. Few studies have examined the relationship between intensity of herbivory and diversity of DTs [4,30,38]. Adams *et al*. [56] hypothesized that intensity of herbivory and DT diversity are positively related because more frequent herbivory could lead to more opportunities for novel DTs to appear. Taking an approach also used by various other authors [8,31], Adams and colleagues used the proportion of damaged leaves, rather than the proportion of leaf area removed, as a measure of the intensity of herbivory; this measure has also been referred to as the ‘frequency' of damage [57]. Adams *et al.* [56] found that present-day high- and mid-latitude sites have similar proportions of damaged leaves but differences in DT diversity, such that no relationship exists between DT diversity and the intensity of insect herbivory.

Previous studies have focused on differences in herbivorized surface area between sites [58], differences in DT diversity and frequency of attack between sites [59,60], or the differences in surface area removal within functional feeding groups—i.e. margin feeding, skeletonization, galling or leaf mining—but not damage-type diversity *per se* [61–63]. To our knowledge, no studies have explicitly addressed possible correlations between the intensity of herbivory, measured by the proportion of leaf area removed, and the diversity of feeding behaviours, measured with counts of FFGs and/or DTs. The lack of clarity surrounding the relationship between these two metrics necessitates further research [55].

### 2.4. The Lower Permian of Texas

Of the series of floras that have been examined worldwide for insect herbivory, the Lower Permian of Texas provides an especially rich opportunity to examine the effects of sampling. Of the sites examined thus far from the Lower Permian of Texas, the first was published before the introduction of the DT system [26], and for the second [27], DT data from individual plant specimens are not available. However, for the third and fourth sites that have been published, Colwell Creek Pond (CCP) and Mitchell Creek Flats (MCF), qualitative and quantitative data are available for each individual plant specimen [5,28]. In addition, CCP and MCF are both mid-Cisuralian in age and have similar dominant broadleaf plant hosts: *Taeniopteris* Brongniart and gigantopterids account for two of the three dominant plant hosts at CCP and are the two dominant plant hosts at MCF.

Here, data from CCP and MCF will be used to examine the impact of sampling effort on quantitative and qualitative measures of insect herbivory and to evaluate subsampling methods that have the potential to reduce the bias of sampling effort in measures of herbivory. These results will facilitate more meaningful comparisons of insect herbivory in deep time.

## 3. Material and methods

All analyses presented here are based on quantitative data—the total and herbivorized leaf area—and qualitative data—the number of DTs observed—for individual broadleaf plant specimens from CCP and MCF. These per-specimen datasets were not included in the original publications, but are presented here as electronic supplementary material, table S1. Our analysis is based on only two localities because these are the only localities for which specimen-by-specimen datasets are available to date with both quantitative and qualitative data.

The analyses here focus on broadleaf taxa only. Other types of plants and plant organs—such as conifer needles, axes, fructifications and seeds—are present at the sites examined from the Permian of Texas, but have greatly reduced diversity of herbivory, with between zero and three DTs noted for each [5,28]. In addition, the proportion of specimens belonging to broadleaf taxa varies greatly from locality to locality and, therefore, can strongly bias between-locality comparisons. For this reason, some previous between-locality comparisons of raw herbivory data included broadleaf taxa only [5,28].

At both sites, a few taxa account for the vast majority of broadleaf specimens. These are referred to here as the ‘dominant' plant hosts. CCP is dominated primarily by the peltasperm *Auritifolia waggoneri* Chaney, Mamay, DiMichele & Kerp and the polyphyletic form taxon *Taeniopteris* Brongniart, and secondarily by the gigantopterid *Evolsonia texana* Mamay. MCF is dominated primarily by *Taeniopteris* spp. and secondarily by an indeterminate species in the gigantopterid genus *Zeilleropteris* Mamay. *Evolsonia texana* is the only gigantopterid present at CCP, but a total of four discrete gigantopterid taxa have been recognized at MCF. A ‘gigantopterid' category, consisting of *Zeilleropteris* sp. and all of the rarer gigantopterids at MCF, was also analysed here as a secondarily dominant plant host.

An ‘indeterminate broadleaf' category was used at both CCP and MCF. However, plants in this category are not analysed here because this category includes poorly preserved specimens belonging to various broadleaf taxa. Whereas *Taeniopteris* is a form genus, the ‘indeterminate broadleaf' category is a wastebin designation. All specimens belonging to *Taeniopteris*, whether ferns or cycadophytes, share distinctive morphological characteristics that would similarly influence patterns of insect herbivory, but the same is not true of the specimens assigned to the morphologically disparate ‘indeterminate broadleaf' category. Seventy-four leaves have been assigned to the ‘indeterminate broadleaf' category at CCP; other discrete broadleaf plant hosts not considered to be ‘dominant’ are represented by between 1 and 32 specimens at CCP and MCF. At MCF, none of the non-dominant broadleaf plant hosts include multiple specimens with insect damage, and so none of the non-dominant broadleaf plant hosts are analysed here.

All analyses were conducted in R v. 3.3.2 [64] and all figures were created with the R package ggplot2 v. 2.2.1 [65].

Many analyses of insect herbivory in the fossil record do not include leaf area, presumably because measuring this variable is time-consuming. We, therefore, tested how many leaves per taxon per site must be measured in order for the subsampled total leaf area to approximate the true value. We conducted this analysis for the three most abundant plant hosts: *Taeniopteris* spp. at MCF and CCP, and *A. waggoneri* at CCP; total leaf area has already been measured for each of these taxa. First, the 95% confidence interval for mean leaf area was calculated using a bootstrapping procedure that was repeated 10 000 times. Then secondly, leaf area measurements for each taxon were subsampled in intervals of 10 specimens—at 10, 20, 30, etc. specimens. Each subsampling routine was also repeated 10 000 times and the 95% confidence interval was then calculated for each interval.

Our analysis of quantitative data focuses on the herbivory index: the percentage of total surface area damaged by insect herbivory. The herbivory index has previously been used to compare the intensity of insect feeding from the Permian of Texas [5]. For each site and for each dominant plant host, 95% confidence intervals were calculated from 5000 replicate bootstrapping runs. The herbivory index was then compared to the number of specimens drawn and the total amount of leaf surface area sampled.

Our analyses of qualitative data focus on total diversity of DTs per plant host. We computed rarefaction curves of DT diversity against number of leaves sampled using the Bernoulli product model for sample-based interpolation [66]. To calculate confidence intervals, we used replicate bootstrapping runs, following previous studies [67], with 5000 replicates. We display our curves with 84% confidence intervals, following established reasoning for rarefied diversity data: for the comparison of two curves, 84% confidence intervals will yield a Type I error rate of less than 0.05 [68].

Both the herbivory index and rarefied DT diversity were calculated from two datasets per locality/plant host. In the first dataset, called the ‘all-specimen' dataset, all specimens were included in the analyses regardless of whether they were damaged by insect herbivores. In the second dataset, called the ‘damaged-only' dataset, the only specimens included in the analyses were those that had been damaged by insect herbivores.

Differences in leaf size distribution between sites were analysed with a Kruskal–Wallis rank sum test, using the base-R function kruskal.test().

We compared correlations between the three most common metrics of the intensity of insect herbivory: the herbivory index (the proportion of leaf area damaged by insects), the proportion of leaves herbivorized and DT diversity. We conducted these comparisons on raw and subsampled data for both localities and for the primarily dominant plant hosts. Correlations between the three metrics of herbivory, outlined above, were compared using pairwise *R*-squared values. For CCP, whole-locality data were subsampled at 500, 250 and 100 leaves, and data from *Taeniopteris* spp. and *A. waggoneri* were subsampled at 250, 100 and 50 leaves. For MCF, which contains far fewer specimens, whole-locality data were subsampled at 100 leaves and *Taeniopteris* spp. was subsampled at 50 leaves. Each subsampling routine was repeated 1000 times.

## 4. Results

**Figure 2.**
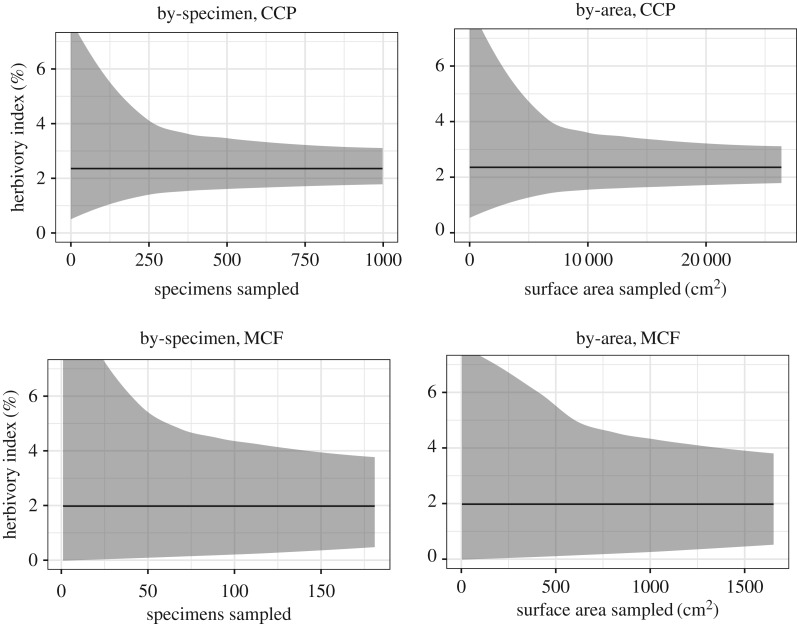
Subsampled herbivory indices for all broadleaf plants from CCP and MCF. Based on the ‘all-specimens' dataset. The mean is in black and the 95% confidence interval is in grey.

**Figure 3.**
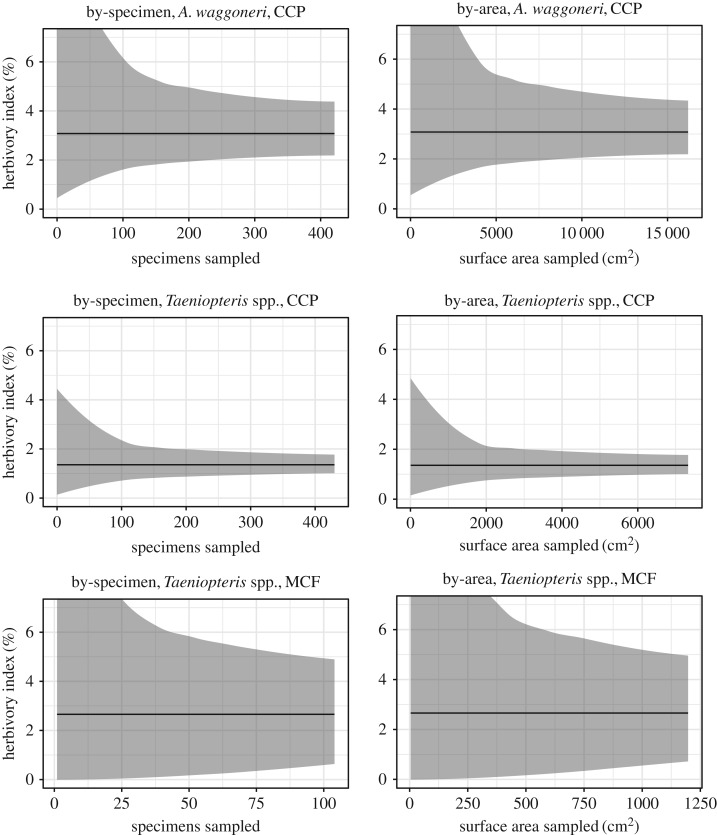
Subsampled herbivory indices for the primary dominant plant hosts from CCP and MCF. Based on the ‘all-specimens' dataset. The mean is in black and the 95% confidence interval is in grey.

The herbivory index is highly variable, both for entire sites (figure 2) and for individual plant hosts (figure 3; electronic supplementary material, figure S1). The primarily dominant plant host at MCF, *Taeniopteris* spp., is represented by 104 specimens and 1197.43 cm^2^ of surface area; when *Taeniopteris* spp. from CCP is subsampled at this amount, the 95% confidence interval ranges from 0.74% to 2.24% with by-specimen subsampling, and from 0.64% to 2.53% with by-area subsampling (figure 3). When *A. waggoneri* from CCP is subsampled at this amount, the 95% confidence interval ranges from 1.68% to 5.87% with by-specimen subsampling, and from 1.12% to 9.19% with by-area subsampling (figure 3). When the herbivory index is bootstrapped for secondarily dominant plant hosts, which are represented by fewer than 100 specimens each, the herbivory index is even more highly variable (electronic supplementary material, figure S1).

**Figure 4.**
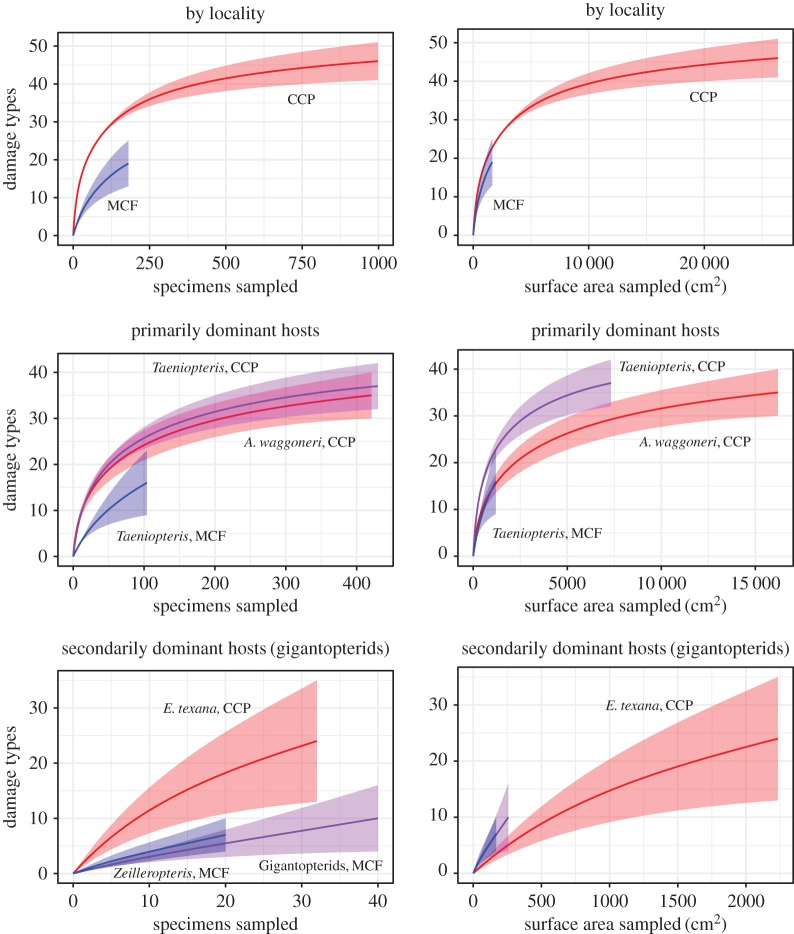
Rarefied DT diversity, by locality and plant host. Based on the ‘all-specimens’ dataset. The lines represent the mean values and the shaded areas represent 95% confidence intervals.

The difference in leaf surface area between CCP and MCF is highly significant (*p* < 0.0001), but is not significant between individual plant hosts from the two localities (electronic supplementary material, table S2; figure S6). When subsampled DT diversity is compared between different plant hosts or localities, the resulting patterns change depending on whether DT diversity is plotted against the number of specimens sampled or the amount of surface area sampled (figure 4).

When DT diversity is subsampled for entire localities, by-specimen rarefaction curves show CCP as clearly having higher DT diversity than MCF. However, when curves are corrected for surface area sampled, DT diversity is nearly indistinguishable for the two localities (figure 4). For individual plant hosts, as for entire localities, patterns of DT diversity change when subsampling results are corrected for differences in surface area.

The results discussed above were calculated with the ‘all-specimens' dataset. When the ‘damaged-only' dataset is used, the results reported above still hold: the herbivory index is highly variable (electronic supplementary material, figures S2, S3, S4), and comparisons of DT diversity change depending on whether DT diversity is plotted against number of specimens or surface area (electronic supplementary material, figure S5). However, the relative herbivory indices change depending on whether the ‘all-specimens' or ‘damaged-only' dataset is used (table 1). According to the ‘all-specimens' dataset, CCP has a higher herbivory index than MCF (2.36% versus 1.98%; table 1). But based on the ‘damaged-only' dataset, MCF has a noticeably higher herbivory index than CCP (4.22% versus 2.71%; table 1), though the confidence intervals for the two localities still overlap (table 1; electronic supplementary material, figure S2).

**Table 1.** The herbivory index from each locality and plant host, calculated from the ‘all-specimens' and ‘damaged-only' datasets. The lower limit of the 95% confidence interval, the mean value and the upper limit of the 95% confidence interval are presented.

locality/plant host	‘all specimens’ (%)			‘damaged only’ (%)		
Colwell Creek Pond (CCP)	1.79	2.36	3.14	2.06	2.71	3.60
Mitchell Creek Flats (MCF)	0.49	1.98	3.74	1.20	4.22	7.78
*Auritifolia waggoneri* (CCP)	2.21	3.08	4.39	2.33	3.28	4.56
*Taeniopteris* spp. (CCP)	1.00	1.35	1.75	1.30	1.77	2.32
*Taeniopteris* spp. (MCF)	0.76	2.66	5.20	1.67	5.39	9.14
*Evolsonia texana* (CCP)	0.45	0.95	1.73	0.55	1.10	2.07
*Zeilleropteris* sp. (MCF)	0.09	0.36	0.72	0.41	1.16	2.63
all gigantopterids (MCF)	0.10	0.32	0.59	0.47	1.11	2.06

Among the primarily dominant plant hosts, *A. waggoneri* and *Taeniopteris* spp. from CCP appear to have nearly indistinguishable DT diversities with by-specimen sampling, with *Taeniopteris* spp. from MCF appearing to have far lower DT diversity (figure 4). But again, when curves are corrected for surface area sampled, DT diversity from MCF falls within the range of DT diversity from CCP, and differences become apparent in DT diversity for *A. waggoneri* and *Taeniopteris* spp. from CCP. Leaf surface area of primarily dominant plant hosts is not significantly different between the two localities (electronic supplementary material, table S2; figure S6).

Among the secondarily dominant plant hosts, apparent patterns of herbivory are reversed between CCP and MCF depending on whether DT diversity is plotted against specimens sampled or surface area sampled. With by-specimen sampling, confidence intervals for gigantopterids from CCP and MCF diverge after only 15 specimens have been sampled, with DT diversity for *E. texana* from CCP higher than that of the MCF plant hosts (figure 4). When rarefaction curves are corrected for surface area sampled, the confidence intervals for the three gigantopterids overlap and DT diversity appears higher at MCF than at CCP. Leaf surface area of secondarily dominant plant hosts is significantly different between the two localities (electronic supplementary material, table S2).

**Figure 5.**
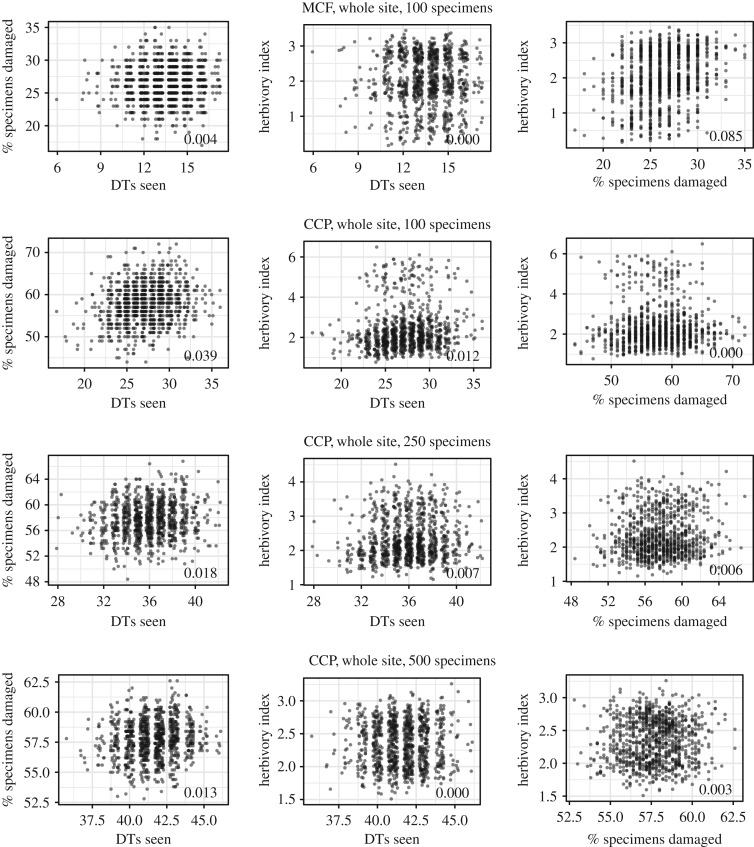
Comparisons of subsampled DT diversity, the herbivory index, and proportion of leaves damaged, by locality. Based on the ‘all-specimens' dataset. *R*-squared values are noted in the bottom right corner of each plot.

Regardless of the extent to which data are subsampled, there is almost no correlation—and certainly no statistically significant correlation—between the three common measures of fossil insect herbivory: DT diversity, herbivory index and the proportion of leaves damaged (figure 5, electronic supplementary material, figure S7, S8).

**Figure 6.**
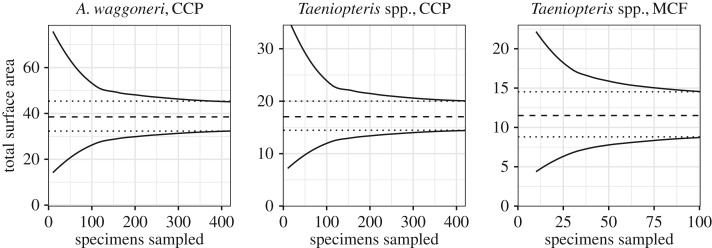
Total leaf area. The dashed lines represent the mean for the complete dataset, the dotted lines represent the 95% confidence interval for the complete dataset, and the solid lines represent the 95% confidence interval for subsampled datasets.

When leaf area is measured for a subsampled portion of the available data, the subsampled confidence intervals are notably wider than the true confidence intervals until sampling is nearly exhaustive (figure 6). For *Taeniopteris* spp. from MCF, for which little more than 100 specimens are available, the confidence intervals narrow when 30–40 specimens have been subsampled. For *Taeniopteris* spp. and *A. waggoneri* from CCP, which are both represented by over 400 specimens, the confidence intervals narrow notably when over 100 specimens have been sampled, and there is a negligible difference in width between the subsampled and true confidence intervals when over 300 specimens have been sampled.

## 5. Discussion

The results presented here demonstrate the necessity of sampling standardization for studies of insect herbivory in deep time. Our findings indicate that (i) sampling standardization, or lack thereof, can strongly influence comparisons of herbivory; (ii) leaf surface area must be taken into account when comparing insect herbivory; (iii) all specimens, herbivorized or not, should be included in rarefaction analyses; and (iv) the herbivory index, the proportion of damaged leaves, and DT diversity are not correlated and therefore cannot be used as a proxy for each other. We conclude by providing suggestions for sampling standardization in future herbivory studies.

### 5.1. The herbivory index

Our results show that the herbivory index is highly variable for well-sampled taxa, even when over 100 specimens and over 1000 cm^2^ of surface area have been sampled (figure 2). This finding suggests that comparisons of the herbivory index should always include confidence intervals. Previous studies have used the herbivory index, with no confidence intervals presented, to compare herbivory at different localities [5,28]. The results presented here suggest that the herbivory index should be interpreted only within the context of confidence intervals.

Because of the wide confidence intervals recovered here, we suggest that future studies present subsampled herbivory indices with 95% confidence intervals for each individual taxon, calculated for a wide range of values such as 100, 250 and 500 specimens and at 1000, 2500 and 5000 cm^2^ of leaf surface area. Assuming that the MCF and CCP datasets are typical of most such studies of plant–insect interactions, the results presented here suggest that the herbivory index should not be calculated for fewer than 100 specimens, or for less than 1000 cm^2^ of surface area, because the wide confidence intervals at this level of sampling preclude meaningful comparisons.

### 5.2. Damage type diversity

None of the rarefaction curves in this study have reached an asymptote (figure 4). This suggests that exhaustive sampling of hundreds of leaves still does not provide a complete view of the DT diversity at a given locality or for a given plant host at localities analogous to MCF or CCP. Because corrections for leaf surface area cause dramatic differences in relative patterns of herbivory—even for plant groups whose specimen sizes are not significantly different, such as *A. waggoneri* from CCP and *Taeniopteris* spp. from MCF—it is clearly essential for all estimates of DT diversity to be compared in the context of leaf surface area.

When DT diversity is rarefied, results are typically presented only for a particular number of leaves sampled [4,8,11–13,69,70]; complete rarefaction curves are rarely published (e.g. [19,36]). Our results show the value of presenting complete rarefaction curves, rather than rarefying to a single, predetermined number of specimens or amount of leaf surface area. Among the secondarily dominant plant hosts, *Zeilleropteris* sp. from MCF has a higher diversity of DTs than *Evolsonia texana* from CCP. However, *Zeilleropteris* sp. is represented by only 168 cm^2^ of surface area; at this low level of sampling, the differences in DT diversity between primarily dominant plant hosts, and between entire localities, are not clear. The presentation of complete rarefaction curves, therefore, allows recognition of patterns of DT diversity regardless of the sampling threshold at which differences in DT diversity can be recognized.

### 5.3. Criteria for inclusion of leaves

The two methods for calculating the herbivory index—including all leaf specimens (the ‘all-specimens’ dataset), or including only leaf specimens with insect damage (the ‘damaged-only' dataset)—yield conflicting results. The ‘all-specimens' dataset shows that CCP has a marginally higher herbivory index than MCF (figure 2), whereas the ‘damaged-only' dataset shows that the herbivory index of MCF is nearly twice that of CCP (electronic supplementary material, figure S2). This discrepancy probably arises from the size of leaf fragments at the two sites: the average *Taeniopteris* specimen has a surface area of 17.04 cm^2^ at CCP but only 11.51 cm^2^ at MCF, and the average gigantopterid specimen has a surface area of 69.83 cm^2^ at CCP but only 6.44 cm^2^ at MCF. There is no evidence to suggest that the leaves at CCP were larger than those at MCF; rather, the available specimens are all fragmentary, and leaves appear to have been broken up into more and smaller fragments at MCF.

If two identical sets of leaves are broken into different sized fragments, this fragmentation can bias measurements of the herbivory index if only the damaged specimens are included in analyses. For example, if two sites, Site A and Site B, contain 100 leaves each, and if each leaf measures 10 cm^2^ and has a single damaged area that measures 0.1 cm^2^, both sites should have the same herbivory index. This result will be recovered if all specimens are counted or if the leaves from each site are fragmented to the same extent during the fossilization and collection processes. However, if the leaves from Site A are broken into an average of two fragments, and if the leaves from Site B are broken into an average of four fragments, calculation of the herbivory index with the ‘damaged-only' dataset will incorrectly portray Site A as having an herbivory index twice as high as that of Site B. This will occur because undamaged fragments will be incorrectly assumed to have come from undamaged leaves.

For comparisons of the herbivory index, the ‘all-specimens' dataset is robust to differences in leaf fragment size, whereas the ‘damaged-only' dataset is not. For this same reason, the ‘damaged-only’ dataset would not yield comparable herbivory indices for sites whose leaves have been sampled according to different criteria, e.g. 50% or greater of the blade intact versus 0.5 cm^2^ or greater in area. For this reason, and for the reasons mentioned in the Introduction, we recommend the inclusion of all specimens, damaged and undamaged, in comparisons of insect herbivory.

### 5.4. Correlations between metrics

The herbivory index, DT diversity and the proportion of herbivorized leaves are uncorrelated in all comparisons conducted here. This is not surprising given that each of these metrics measures a different aspect of plant–insect associational ecology and there are likely to be pronounced differences between herbivore diversities and intensities at modern study sites. There was no significant correlation between these common metrics of diversity when both raw data and subsampled data were analysed. These findings indicate that no metric of herbivory can be used as a proxy for the others and that, for a complete comparison of insect herbivory, both DT diversity and the herbivory index should be measured.

### 5.5. Guidelines for the study of insect herbivory in deep time

Deep-time insect herbivory research would benefit from standardization of collecting, sampling and analysing techniques. Our current ability to compare localities across time and space has been greatly hampered by this lack of standardization. Here, we propose guidelines for excavating quarries, sampling floras and collecting and analysing data. These guidelines fall into two categories: definitive guidelines to be followed in all future studies, and more tentative guidelines to be evaluated with data from additional localities and intervals.

— Primary guidelines
  ○ Collecting and sampling procedures should be explicitly stated, with particular attention to the biases outlined above in the ‘Sampling of fossil leaves' section.
  ○ Whole-locality metrics should be calculated for broadleaf plants only, in order to control for varying proportions of microphyll leaves, seeds, axes, etc. across different localities.
  ○ Whenever possible, three metrics should be reported: the herbivory index, proportion of herbivorized leaves and DT diversity. None of these metrics cannot be used as a proxy for another, and together they provide a more holistic assessment of herbivory.
  ○ All metrics should be reported for entire localities and for the dominant broadleaf plant hosts at each locality.
  ○ If all specimens above a certain size threshold are studied, the above metrics should also be calculated on a subset of the data that only includes leaves for which 50% or more of the leaf area is present.
  ○ Confidence intervals should be presented for all measures of herbivory.
  ○ Rarefaction curves should be scaled by total leaf area. Total leaf area should be measured exhaustively or for at least 300 specimens per plant host per site.
  ○ Complete rarefaction curves should be presented, rather than the mean and variance for rarefaction to a single, predetermined number of specimens.
  ○ The herbivory index and rarefied DT diversity should both be calculated from the ‘all-specimen' dataset.
  ○ In the light of the results presented by Gunkel & Wappler [8], floral diversity and evenness should be taken into consideration when comparing insect herbivory between localities.
  ○ Also in the light of the results presented in the recent contribution cited above [8], the degree of specialization associated with each DT should be taken into account.
  ○ A specimen-by-specimen dataset—including total leaf surface area, herbivorized surface area and presence/absence of DTs—should be made available, as supplementary material or an appendix, so that future researchers can further analyse the data.
  ○ Findings based on a single metric should not be presented in terms of ‘increased herbivory' or ‘decreased herbivory,' because both herbivorized surface area and DT diversity have been used as proxies for total ‘herbivory' but these two metrics are uncorrelated with one another. Terminology such as ‘increased herbivory’ and ‘decreased herbivory' should be used with caution, perhaps reserved for instances in which both metrics are in agreement.
— Auxiliary guidelines
  ○ Ideally, at least one host plant per site should be represented by at least 250 specimens. However, a minimum of 100 specimens from a dominant host plant may be sufficient.
  ○ Each site should ideally contain at least 1000 specimens, with at least 200 broadleaf specimens.

### 5.6. Future directions

The data analysed here represent only two localities because these are the only two localities for which the necessary datasets (qualitative and quantitative data for each specimen) are available. Future studies that include both qualitative and quantitative data can be used to refine the conclusions presented here.

These conclusions can be expanded and refined in three major ways. Firstly, future studies can test the relevance of our results to other types of floras. At a broad scale, comparisons can be made between older floras that are dominated by lycopods, ferns, horsetails and gymnosperms, with more recent floras that are dominated by angiosperms. At a finer scale, comparisons of herbivory between plant assemblages from a variety of sedimentary facies, even within a single member of a formation, may demonstrate the effect of changing palaeoenvironments. There are often differences in species diversity, frequency, productivity and other measures of variation, which can alter the strength and likelihood of plant–herbivore interactions [71,72]. A recent contribution [8] evaluated the importance of floral diversity and evenness when comparing herbivory at different localities and evaluated the importance of rare and specialized DTs; future studies can evaluate the impact of floral evenness and DT rarity when the amount of leaf surface area examined is accounted for.

Furthermore, the thresholds tentatively suggested here, for the minimum number of leaf specimens per site and for the dominant plant host, are based on our findings from CCP and MCF. These thresholds may not apply to other localities, for two main reasons. Firstly, CCP and MCF are dominated by *Taeniopteris* spp., a form taxon that is definitely polyphyletic at CCP and quite possibly polyphyletic at MCF; most other localities, including some from the late Palaeozoic of Texas, are dominated by plant hosts that are believed to be monophyletic [26,27]. In addition, angiosperm-dominated ecosystems differ from gymnosperm-dominated ecosystems in multiple ways. They contain a higher diversity of niches [73] and appear to be more diverse overall. Also, angiosperm leaf architecture and productivity is fundamentally different from that of gymnosperms [74,75]. These basic differences raise the question of whether confidence intervals for measures of insect herbivory on angiosperm leaves may be of different widths—which, again, could change the thresholds suggested here. Future studies should, therefore, revisit the thresholds presented here, by calculating confidence intervals for all metrics of herbivory.

Secondly, future studies can explore additional factors that necessitate sampling standardization. An important sampling method that must be further analysed is the surface area of sampled leaves. The insignificant differences in leaf area recovered here for individual plant hosts may be an artefact of the sampling method used; the inclusion of many small fragments may obscure significant size differences among intact leaves. This practice may have obscured true differences between plant hosts by artificially reducing the lower bound of each host's leaf area. For this case study, however, it was impossible to analyse only those leaves that are at least 50% complete because many of the available leaf specimens are fragments that are missing both the base and apex, making it impossible to determine whether the fragment represents more or less than 50% of the original leaf area. Under different sampling regimes, in which the only leaves collected are those that are at least 50% complete, it may not be necessary to correct for surface area among plant hosts with insignificant differences in total surface area.

Lastly, the results presented here suggest that DT diversity and the herbivory index are decoupled, but our results do not indicate why this is the case. Future studies can explore the underlying factors that cause DT diversity and the herbivory index to diverge; an understanding of this phenomenon, perhaps using neontological data, would facilitate more nuanced interpretations of the data that are currently available and could guide future sampling. Wappler and colleagues [70,76] examined changes in DT diversity within individual FFGs, an important first step that can be implemented without changing the way data are collected. Adams and colleagues [58,61,62] measured the percentage of leaf area damaged by herbivores separately for four FFGs. These measurements were not accompanied by counts of DT or FFG diversity, and, therefore, cannot be used to directly address the question at hand. However, the strategy of measuring the herbivory index separately for each FFG or DT would almost certainly aid in disentangling the relationship between DT diversity and the herbivory index.

## Supplementary Material

Supporting Information

## Supplementary Material

Raw data

## Data accessibility

All data used for the analyses here are available as a supplemental .csv file.
