# Supplementary material for: The importance of sampling standardization for comparisons of insect herbivory in deep time: a case study from the late Palaeozoic
Source: R Soc Open Sci. 2018 Mar 28;5(3):171991. doi: 10.1098/rsos.171991 (PMC5882722; doi:10.1098/rsos.171991)
Supplement: Supporting Information [file rsos171991supp1.docx]

## Supporting Information

Article title: The importance of sampling standardization for comparisons of insect herbivory in deep time: a case study from the late Paleozoic

Authors: Sandra R. Schachat, Conrad C. Labandeira, S. Augusta Maccracken

The following Supporting Information is available for this article:

**Fig. S1** Subsampled herbivory indices for the secondarily dominant plant hosts from CCP and MCF (gigantopterids). Based on the “all-specimens” dataset.

**Fig. S2** Subsampled herbivory indices for all broadleaf plants from CCP and MCF. Based on the “damaged-only” dataset.

**Fig. S3** Subsampled herbivory indices for the primary dominant plant hosts from CCP and MCF. Based on the “damaged-only” dataset.

**Fig. S4** Subsampled herbivory indices for the secondarily dominant plant hosts from CCP and MCF (gigantopterids). Based on the “damaged-only” dataset.

**Fig. S5** Rarefied Damage Type (DT) diversity, by plant host. Based on the “damaged-only” dataset.

**Fig. S6** Leaf surface area at CCP and MCF. Based on the “all-specimens” dataset.

**Fig. S7** Comparisons of subsampled DT diversity, the herbivory index, and proportion of leaves damaged, for *A. waggoneri* from CCP. Based on the “all-specimens” dataset.

**Fig. S8** Comparisons of subsampled DT diversity, the herbivory index, and proportion of leaves damaged, for *Taeniopteris* spp. from CCP and MCF. Based on the “all-specimens” dataset.

**Table S1** The raw data used for all analyses here.

**Table S2** *p*-values resulting from the Kruskal-Wallis rank sum test comparing per-specimen surface area at CCP and MCF.

**Fig. S1** Subsampled herbivory indices for the secondarily dominant plant hosts from CCP and MCF (gigantopterids). Based on the “all-specimens” dataset.

**Fig. S2** Subsampled herbivory indices for all broadleaf plants from CCP and MCF. Based on the “damaged-only” dataset.

**Fig. S3** Subsampled herbivory indices for the primary dominant plant hosts from CCP and MCF. Based on the “damaged-only” dataset.

**Fig. S4** Subsampled herbivory indices for the secondarily dominant plant hosts from CCP and MCF (gigantopterids). Based on the “damaged-only” dataset.

**Fig. S5** Rarefied Damage Type (DT) diversity, by plant host. Based on the “damaged-only” dataset.

**Fig. S6** Leaf surface area at CCP and MCF. Based on the “all-specimens” dataset.

**Fig. S7** Comparisons of subsampled DT diversity, the herbivory index, and proportion of leaves damaged, for *A. waggoneri* from CCP. Based on the “all-specimens” dataset.

**Fig. S8** Comparisons of subsampled DT diversity, the herbivory index, and proportion of leaves damaged, for *Taeniopteris* spp. from CCP and MCF. Based on the “all-specimens” dataset.

**Table S1** The raw data used for all analyses here.

Attached as an Excel file, rawdata.csv

**Table S2** *p*-values resulting from the Kruskal-Wallis rank sum test comparing per-specimen surface area at CCP and MCF.

|  |  | Whole site | *Taeniopteris* spp. | *A. waggoneri* | *E. texana* |
| --- | --- | --- | --- | --- | --- |
|  |  | CCP | CCP | CCP | CCP |
| Whole site | MCF | <0.0001 |  |  |  |
| *Taeniopteris* spp. | MCF |  | 0.3340 | 0.6382 |  |
| *Zeilleropteris* sp. | MCF |  |  |  | 0.0386 |
| Gigantopterids | MCF |  |  |  | 0.0001 |
